# Translation, cultural adaptation, and validation of the Genital Self-Image Scale (GSIS-20) for the Portuguese language

**DOI:** 10.1590/1806-9282.20250590

**Published:** 2026-09-28

**Authors:** Denise Saquetto, Renata Almeida de Souza Aranha e Silva, Aline Marina Marcondes Braga Rosa, Mariane Castiglione, Thaiane Suleiman Saoud, César Eduardo Fernandes, Luiz Gustavo Oliveira Brito, Emerson Oliveira

**Affiliations:** 1 Centro Universitário Faculdade de Medicina do ABC, Department of Urogynecology and Pelvic Floor Dysfunction, Discipline of Gynecology – Santo André (SP), Brazil.; 2 Universidade de Campinas, School of Medical Sciences, Department of Obstetrics and Gynecology – Campinas (SP), Brazil.

**Keywords:** Female genital, Pelvic organ prolapse, Validation study, Translation, Quality of life

## Abstract

**OBJECTIVE:**

The aim of this methodological study was to translate, culturally adapt, and validate the Genital Self-Image Scale (GSIS-20) into Brazilian Portuguese.

**METHODS:**

The cross-cultural adaptation followed a standardized five-step process: (1) initial translation into Portuguese by two independent bilingual translators; (2) synthesis of the translations; (3) back-translation into English by two independent native speakers; (4) expert committee review to ensure semantic, idiomatic, experiential, and conceptual equivalence; and (5) pre-test with 15 women to evaluate comprehension and cultural relevance. Subsequently, the psychometric properties of the adapted instrument were assessed in 148 women divided into three groups: nulliparous (n=53), primiparous without pelvic organ prolapse (n=52), and women with stage III or IV pelvic organ prolapse (n=43). The Genital Self-Image Scale (GSIS-20) was applied at three time points to assess internal consistency (Cronbach's alpha), test–retest, and interobserver reliability (intraclass correlation coefficient), and discriminant validity.

**RESULTS:**

All subscales of the Genital Self-Image Scale (GSIS-20) showed statistically significant differences between groups. The instrument demonstrated excellent internal consistency (Cronbach's alpha=0.925) and reproducibility (intraclass correlation coefficient >0.75).

**CONCLUSION:**

The Genital Self-Image Scale (GSIS-20) was successfully translated and culturally adapted into Brazilian Portuguese, showing satisfactory psychometric properties for use in this population.

## INTRODUCTION

Female genital self-image is defined as the perception of one's own genitalia, crucial to female sexuality from a biopsychosocial standpoint^1,2^. Positive genital and body self-image improves quality of life. Women with a positive genital self-image exhibit higher self-esteem, sexual confidence, and quality of life while feeling secure in seeking gynecological care. Conversely, negative perceptions can lead to anxiety, depression, sexual dysfunctions, and pursuit of aesthetic procedures^2-7^.

Many women increasingly question their genital appearance, often associating a negative vulva image with issues like odor, size, and shape, impacting sexual quality of life^6^. Body and genital image issues may be overlooked during pregnancy, postpartum, or with pelvic dysfunctions like urinary incontinence or pelvic organ prolapse, adversely affecting sexual function^4^. Surgical interventions to address these dysfunctions can enhance sexual satisfaction by improving body and genital image^8^.

There is a growing demand for surgical procedures to enhance genital morphology, self-esteem, functional comfort, and sexual pleasure, with cosmetic gynecology expanding rapidly as women seek the "ideal" vulva^3^. Common treatments, albeit not evidence-based, include cosmetics, piercings, and female cosmetic genital surgery^5^. Media influence is significant in these trends, with labiaplasty being prevalent for appearance or comfort concerns^3^. Non-surgical options like laser and radiofrequency are rising, requiring more evidence^9^. These issues call for an interdisciplinary approach considering psychological aspects^10^.

The Genital Self-Image Scale (GSIS) was originally developed in the United States by Berman et al. in 2003 to evaluate women's perceptions of their genitalia as an essential component of sexual health^1^. The original version comprised 29 items, which was later shortened and psychometrically validated in a 20-item version (GSIS-20) by Zielinski et al. in 2012^7^. The GSIS-20 assesses four distinct domains: genital confidence, appeal, function, and comfort. Each domain is scored using a four-point Likert scale, with reverse scoring applied to positively phrased items. Despite its validation in Turkey, the United States, and Egypt^4,11,12^, there is no culturally adapted version for Brazilian Portuguese. Given that instruments developed in different linguistic and cultural contexts may not be directly applicable elsewhere, cross-cultural adaptation and psychometric validation are essential steps before use in new populations.

## METHODS

### Study design and population

We conducted a cross-sectional study from February to December 2018, approved by the Institutional Review Board of the Centro Universitário FMABC (approval number 2,418,543). The sample included 151 women, divided into three groups: 56 nulliparous (GI), 53 primiparous without Pelvic Organ Prolapse (POP) (GII), and 56 with stage III and IV POP (GIII), assessed by the POP-Q^13^. Participants, aged 25–80, were evaluated by the Urogynecology and Pelvic Floor team at Centro de Atenção Integral à Saúde da Mulher, São Bernardo do Campo, São Paulo, Brazil, an affiliated institution with Faculdade de Medicina do ABC (FMABC).

Inclusion criteria were consent to participate, biological female sex, female gender identity, and literacy. Exclusions were pregnant women and those with prior POP or labiaplasty surgeries.

### Translation and validation of the instrument

The GSIS-20, developed by Berman et al.^1^ and validated by Zielinski et al.^7^, was used. Authorization was obtained from Professor Laura Berman for translation and validation. The GSIS-20 assesses four domains: genital confidence, appeal, function, and comfort. Scores range from 0 to 40, with higher scores indicating greater satisfaction. Items are on a Likert scale from 0 to 3, and items with positive words are reversed.

The guidelines by Beaton et al.^14^ guided the translation process. It included translation into Portuguese, cultural adaptation by a medical team for consensus (>50% agreement), back-translation into English to compare with the original, and a pilot study with 30 patients for feedback on comprehensibility.

### Instrument and procedures

Post-informed consent and medical visit, sociodemographic data were collected. Participants self-administered the GSIS-20 without clarification, completing it at T0 (after the visit), T1 (15 min after T0), and T2 (15 days later), overseen by researchers Denise Saquetto and Aline Marina Marcondes Braga Rosa. Measures included internal consistency, test-retest, and interobserver reliability. Internal consistency assesses item correlation; a retest occurred 15 days after the first assessment. Interobserver reliability was checked for 15 min post-interview, while discriminant validity involved applying the questionnaire to all three groups.

Cronbach's alpha assessed intraclass correlation coefficient (ICC): >0.9 is excellent; 0.7–0.9 is good; 0.6–0.7 is questionable; 0.5–0.6 is poor; <0.5 is unacceptable^15^. When ICC=0, no reproducibility exists; ICC=1 indicates maximum reproducibility; ICC <0.4 is poor; ICC≥0.75 is excellent; and 0.4≤ICC<0.75 is satisfactory^16^.

### Statistical analysis

Data were tabulated in Google Drive spreadsheets, analyzed using Prism 8.4.1 and Statistical Package for the Social Sciences. Normality was assessed with the Kolmogorov-Smirnov test. Analysis of Variance (ANOVA), Kruskal-Wallis, and Dunn tested continuous variables, while chi-squared tested categorical variables, with all tests two-tailed and a 5% significance level. Sample size was not calculated due to variability in suggested formulas for translation and validation studies^17^. Reliability was confirmed with ICC for comparisons at T0 and T2 (intraobserver) and T0 and T1 (interobserver)^16^.

## RESULTS

We included 53 nulliparous women (GI), 52 primiparous women (GII), and 43 multiparous women with stage III or IV POP (GIII), totaling 148 patients recruited during the study period. We had to exclude 14 women from the study (due to having undergone surgeries for POP correction and/or labiaplasty) during the recruitment period. Demographic data are described in Table 1. Women with POP had a higher mean age than those in GII and GI (64.86±8.97 vs. 37.12±5.54 and 35.04±6.34 years, respectively; p<0.0001), as well as a higher body mass index (BMI) (26.92±3.63 6 kg/m^2^; p=0.028), a greater proportion of married women (86%; p<0.0001), and higher parity [3 (1–10); p<0.001] than other groups. The rate of hypertension was higher among women with POP 60.6% (p<0.0001) than in the other groups.

**Table 1:** Demographic and clinical baseline characteristics.

Variable		GI (n=53)	GII (n=52)	GIII (n=43)	p
Age^a^		35.04 (6.34)	37.12 (5.54)	64.86 (8.97)	<0.0001
BMI (kg/m^2^)^a^		25.05 (4.17)	26.77 (5.32)	26.92 (3.63)	0.028
Ethnicity^b^					
	White	41 (77.3)	37 (71.1)	26 (60.4)	0.194
	Non-white	12 (22.4)	15 (28.9)	17 (39.6)	
Marital status^b^					
	Married	22 (41.5)	35 (67.3)	37 (86)	<0.0001
	Single	31 (58.5)	17 (32.7)	6 (14)	
Education level^b^					
	Primary education	0 (0)	0 (0)	30 (69.8)	<0.0001
	Secondary education	16 (30.2)	22 (42.3)	11 (25.6)	
	Higher education	37 (69.2)	30 (57.7)	2 (4.6)	
Parity^c^		0	1	3 (1–10)	<0.001
Smoking^b^		4 (7.5)	3 (5.8)	2 (4.6)	0.8343
Diabetes^b^		2 (3.8)	1 (1.9)	2 (4.6)	0.5761
Hypertension^b^		7 (13.2)	8 (15.4)	26 (60.6)	<0.0001
Vascular disease^b^		4 (7.5)	0 (0)	2 (4.6)	0.1423

a Values presented as the mean and standard deviation. Statistical test: ANOVA.

b Values presented as numbers and percentages. Statistical test: chi-squared.

c Values presented as median and variation. Statistical test: Kruskal-Wallis. BMI: body mass index. GI: nulliparous women; GII: primiparous women; GIII: multiparous women with stage III or IV pelvic organ prolapse (POP).

Table 2 shows the analysis of the GSIS-20 scores among the three groups. All GSIS-20 subscales showed statistically significant differences between women with POP versus nulliparous women and primiparous women without the disease, confirming that the questionnaire presents discriminant validity.

**Table 2:** Discriminant validity of the Genital Self-Image Scale-20 questionnaire between groups.

Questionnaire domain	GI (n=53)	GII (n=52)	GIII (n=43)	p
Genital confidence^a^	10.34 (1.46)	10.02 (1.82)	5.86 (3.80)	<0.0001
Genital appeal^a^	9.67 (2.53)	9.80 (2.52)	4.48 (3.22)	<0.0001
Genital function^a^	9.28 (1.62)	9.00 (1.63)	5.18 (3.67)	<0.0001
Genital comfort^a^	5.41 (0.74)	5.29 (0.72)	4.14 (1.95)	<0.0004
Total score^a^	34.72 (4.84)	34.12 (5.10)	19.67 (10.62)	<0.0001

a Values presented as the mean and standard deviation. Statistical test: Kruskal-Wallis. Multiple comparisons: GIII<GII and GIII<GI. Statistical test: Dunn's multiple comparison test. GI: nulliparous women; GII: primiparous women; GIII: multiparous women with stage III or IV pelvic organ prolapse (POP).

Internal consistency coefficients (Cronbach's alpha) of the GSIS-20 questionnaire are shown in Table 3. Values above 0.70 were found for all domains, and for the overall score, Cronbach's alpha coefficient was 0.925, showing excellent internal consistency. Therefore, the reliability of the instrument was adequate.

**Table 3:** Internal consistency coefficients (Cronbach's alpha), test–retest intraobserver and interobserver reliability of the Genital Self-Image Scale (GSIS-20) questionnaire.

Questionnaire domain	Number of items	Cronbach's alpha	Intraobserver ICC CI	Interobserver ICC CI
Genital confidence	05	0.887	0.965 (0.951–0.974) p=0.0001	0.986 (0.981–0.990) p=0.0001
Genital appeal	06	0.835	0.966 (0.953–0.975) p=0.0001	0.936 (0.912–0.954) p=0.0001
Genital function	05	0.830	0.984 (0.978–0.989) p=0.0001	0.936 (0.910–0.954) p=0.0001
Genital comfort	04	0.734	0.976 (0.967–0.983) p=0.0001	0.939 (0.915–0.956) p=0.0001
Total score	20	0.925	0.988 (0.984–0.992) p=0.0001	0.969 (0.958–0.977) p=0.0001

ICC: intraclass correlation coefficient; CI: confidence interval.

Finally, no differences were observed in the test–retest comparison of the GSIS-20 translated into Brazilian Portuguese in the intra- or interobserver evaluation. The ICC results have shown excellent reproducibility (ICC>0.75) (Table 3). The final translated version is also available with this manuscript (Supplementary Appendix).

## DISCUSSION

Each society has its own culture, habits, rules, so the translation of a quality-of-life questionnaire should be adapted to the culture of the country. It should have a clear language and be a simple tool that people can answer without difficulty. A questionnaire must be so adapted before the instrument is used in clinical practice or research in populations that speak a different language so that a unified evaluation standard is achieved^18^; that's why a pilot phase was performed so that regionalization was considered in this process. A questionnaire that is valid and reliable in a specific language may not be valid and reliable when used in a different population and may fail to reproduce the same findings when used in other scenarios or by different observers^19^.

This study adhered to the methodological framework proposed by Beaton et al.^14^ for the cross-cultural adaptation of self-report instruments. By following all five recommended stages—translation, synthesis, back-translation, expert committee review, and pre-testing—the research ensured the semantic, idiomatic, experiential, and conceptual equivalence of the GSIS-20. These methodological safeguards are fundamental to preserving the validity of psychometric instruments when applied to populations culturally and linguistically distinct from those for which the instrument was originally developed.

The original GSIS questionnaire was validated in 2003 in English, and it had 29 items^1^. In 2012, it was shortened and validated with reliability measures for 20 items, again in English^7^. The findings of the present study demonstrate acceptable psychometric properties of the Brazilian Portuguese version of the GSIS-20, which allows its use in this population. The general understanding of the translated terms was adequate, and only small changes were necessary. There was adequate internal consistency for all domains of the questionnaire, and Cronbach's alpha coefficient showed excellent internal consistency of the overall score. We also observed, in the test–retest comparison of the GSIS-20, that the correlations were comparable to those of the validation process of Zielinski et al.^7^ and Pakpour et al.^20^. The present study also confirmed the usefulness of the GSIS-20 to assess the impact of POP on the genital image of the participants and thereby demonstrated the discriminant validity. We found that GIII had a score 19.67 points lower than GII and GI (p<0.0001).

Our data suggest that dissatisfaction with genital self-image is associated with demographic and clinical characteristics that impair self-perception. Older, less educated, multiparous, and POP women showed greater dissatisfaction with their genitalia. Thus, our results are in line with the study by Zielinski et al., 2012, which showed that women with POP exhibit genital image deterioration^21^.

Dissatisfaction with genital image reduces the frequency of visits to the gynecologist and the compliance with preventive exams. Negative body image is associated with worse genital self-image^22,23^. This dissatisfaction may also be caused by the lack of information regarding the anatomy of the genitals. Yulevitch et al., 2013 reported that the lack of knowledge about genitalia and the lack of sexual education may be caused by the cultural context, rigid education, shame, and taboos that still exist in societies^2^. During the data collection process, our team observed that participants lacked basic information about the anatomy of the genitals. In addition, it is common for physicians to feel uncomfortable asking them about these issues. This lack of communication contributes to the poor genital self-image.

Women need to know that each vulva has its own shape, color, size, symmetry, odor, and amount of hair, and each vulva is "normal" and unique, with its own characteristics. A study developed by Sharp and Tiggemann, presented to the participants images of real and healthy female genitals, in all their diversity, and a video showing the digital changes that corrected the protruding small labia in models for pornographic magazines. The authors concluded that the video significantly increased the perceptions of women about the diversity of the appearance of the genitals and that the volunteers who watched it transmitted their knowledge to help other women^24^.

This study has strengths and limitations. One limitation is that the pre-test sample included only 15 participants, which falls short of the 30 participants recommended by Beaton et al.^14^, potentially limiting the generalizability of the findings. In addition, we did not assess construct validity by comparing the GSIS-20 with other related instruments, which should be addressed in future research. Nonetheless, the GSIS-20 demonstrated excellent internal consistency, reproducibility, and discriminant validity in the Brazilian population, supporting its applicability in both clinical and research contexts.

A recent validation study in Brazil for the Body Image in Pelvic Organ Prolapse Questionnaire did not find association between prolapse staging and genital body image or with sexual function; however, an impaired body image was associated with worse perception of attractiveness and increased risk for sexual dysfunction^25^. Future studies comparing GSIS with other instruments are needed. Responsiveness was another psychometric variable that was not tested. As for strengths, we have tested most of the psychometrics variables, the presence of two examiners applying the instruments, and the high scores that were found among our data. We believe that the GSIS-20 translated into Portuguese in Brazil is an instrument that can be applied in gynecological visits, contributing to the doctor–patient relationship, since it can evaluate female feelings regarding their genitals and thus help identify factors that contribute to sexual dysfunctions.

## CONCLUSION

The GSIS-20 questionnaire was successfully translated and culturally adapted into Brazilian Portuguese following international guidelines. It demonstrated excellent psychometric properties, including high internal consistency, reproducibility, and discriminant validity. The Brazilian version of the GSIS-20 can be reliably used in clinical and research settings to assess women's genital self-image, particularly in the context of pelvic floor disorders.

## Data Availability

The datasets generated and/or analyzed during the current study are available from the corresponding author upon reasonable request.
