# Supplementary material for: Translation, cultural adaptation, and validation of the Genital Self-Image Scale (GSIS-20) for the Portuguese language
Source: Rev Assoc Med Bras (1992). 2026 Sep 28;72(9):e20250590. doi: 10.1590/1806-9282.20250590 (PMC13623368; doi:10.1590/1806-9282.20250590)
Supplement: Supplementary file 1 [file 1806-9282-ramb-72-09-e20250590-Suppl01.docx]

**ESCALA DA AUTOIMAGEM GENITAL – GSIS-20**

(Berman, Berman, Miles, Pollets e Powell, 2003, revisada por Zielinski, Low, Sampselle e Miller, 2012)

INSTRUÇÕES: Leia os itens a seguir e verifique a categoria aplicável aos SEUS pensamentos ou sentimentos sobre seus órgãos genitais (como lábios, vagina, clitóris ou vulva).

**Sinto ansiedade e preocupação ao pensar em como meus órgãos genitais funcionam.**

( ) sempre ( ) frequentemente ( ) às vezes ( ) nunca

**Quando penso em meus órgãos genitais, me sinto humilhada ou constrangida.**

( ) sempre ( ) frequentemente ( ) às vezes ( ) nunca

**Tenho sentimentos positivos e/ou confortáveis sobre a visão dos meus genitais por meu parceiro*.**

( ) sempre ( ) frequentemente ( ) às vezes ( ) nunca

**Quando penso em meus órgãos genitais, sinto tristeza e depressão.**

( ) sempre ( ) frequentemente ( ) às vezes ( ) nunca

**Fico envergonhada e/ou constrangida pelo formato dos meus órgãos genitais.**

( ) sempre ( ) frequentemente ( ) às vezes ( ) nunca

**Fico envergonhada e/ou constrangida pelo odor dos meus órgãos genitais.**

( ) sempre ( ) frequentemente ( ) às vezes ( ) nunca

**Sinto que meus órgãos genitais funcionam do jeito certo*.**

( ) sempre ( ) frequentemente ( ) às vezes ( ) nunca

**Sinto que meus órgãos genitais são atraentes e excitam meu parceiro*.**

( ) sempre ( ) frequentemente ( ) às vezes ( ) nunca

**Sinto que meus órgãos genitais são normais ou similares aos das outras mulheres*.**

( ) sempre ( ) frequentemente ( ) às vezes ( ) nunca

**Gosto dos meus órgãos genitais*.**

( ) sempre ( ) frequentemente ( ) às vezes ( ) nunca

INSTRUÇÕES: Indique se os adjetivos a seguir descrevem ou não seus sentimentos sobre seus órgãos genitais:

**Não atraente.**

( ) aplica-se a mim ( ) não se aplica a mim

**Constrangedor.**

( ) aplica-se a mim ( ) não se aplica a mim

**Atraente***

( ) aplica-se a mim ( ) não se aplica a mim

**Fétido (mau cheiro).**

( ) aplica-se a mim ( ) não se aplica a mim

**Ofensivo.**

( ) aplica-se a mim ( ) não se aplica a mim

**Inadequado.**

( ) aplica-se a mim ( ) não se aplica a mim

**Saudável*.**

( ) aplica-se a mim ( ) não se aplica a mim

**Funcional.**

( ) aplica-se a mim ( ) não se aplica a mim

**Desejável*.**

( ) aplica-se a mim ( ) não se aplica a mim

**Bem-proporcionado.**

( ) aplica-se a mim ( ) não se aplica a mim

*Itens invertidos para fins de pontuação.
